# Notes on the Abrasive Water Jet (AWJ) Machining

**DOI:** 10.3390/ma14227032

**Published:** 2021-11-19

**Authors:** Lucie Gembalová, Libor M. Hlaváč, Sławomir Spadło, Vladan Geryk, Luka Oros

**Affiliations:** 1 Institute of Clean Technologies for Mining and Utilization of Raw Materials for Energy Use, Faculty of Mining and Geology, VSB—Technical University of Ostrava, 17. Listopadu 2172/15, Poruba, 70800 Ostrava, Czech Republic; lucie.gembalova@vsb.cz; 2 Department of Physics, Faculty of Electrical Engineering and Computer Science, VSB—Technical University of Ostrava, 17. Listopadu 2172/15, Poruba, 70800 Ostrava, Czech Republic; 3 Department of Materials Science and Materials Technology, Faculty of Mechatronics and Mechanical Engineering, Kielce University of Technology, al. Tysiąclecia Państwa Polskiego 7, 25-314 Kielce, Poland; sspadlo@tu.kielce.pl; 4 Department of Mining and Geology, Faculty of Mining and Geology, VSB—Technical University of Ostrava, 17. Listopadu 2172/15, Poruba, 70800 Ostrava, Czech Republic; vladan.geryk@vsb.cz (V.G.); lukas.oros@vsb.cz (L.O.)

**Keywords:** abrasive water jet, abrasive material, abrasive particle, particle size, cutting, microscopy

## Abstract

The aim of the research was to investigate changes of abrasive grains on metals observing the kerf walls produced by the Abrasive Water Jet (AWJ). The microscopy observations of the sidewalls of kerfs cut by the AWJ in several metal materials with an identical thickness of 10 mm are presented. The observed sizes of abrasive grains were compared with the results of research aimed at the disintegration of the abrasive grains during the mixing process in the cutting head during the injection AWJ creation. Some correlations were discovered and verified. The kerf walls observations show the size of material disintegration caused by the individual abrasive grains and also indicate the size of these grains. One part of this short communication is devoted to a critical look at some of the conclusions of the older published studies, namely regarding the correlation of the number of interacting particles with the acoustic emissions measured on cut materials. The discussion is aimed at the abrasive grain size after the mixing process and changes of this size in the interaction with the target material.

## 1. Introduction

The Abrasive Water Jet (AWJ) machining, namely cutting, has been researched for almost 40 years. The first research activities were aimed at the introduction of this new machining tool [1], a description of the cutting process itself [2,3], and the preparation of some regression models enabling basic calculations of the AWJ effectiveness in the cutting process [4]. The subsequent studies were focused on various aspects of the machining processes, covering the specific problems of impact on ductile materials [5], the brittle ones [6], turning [7], and milling [8]. However, some new pieces of knowledge are still occurring, as well as the use of new and more precise methods of the AWJ investigation. Nevertheless, the aim of this communication is to support some older results that are still little taken into account when describing the AWJ machining processes. One of these older results, often neglected, is the fact that the abrasive material disintegrates just in the mixing process present in the mixing chamber and the focusing tube of the cutting head (in the case of the injection AWJ creation). The first results focused on this problem were published in the 1990s [9]. The extent of this disintegration, as it was presented in [10], depends on the pressure inside the pump, but also on the size of the orifice and the focusing tube, the original abrasive grain size, type of the abrasive material and its quality. Finally, the abrasive grain size resulting from the mixing process also depends on a construction of the mixing head, as it was investigated in [11]. The specific surface energy is one of the key properties characterizing the abrasive material quality and influencing the rate of its disintegration [12]. Therefore, it should be studied deeper in the future for all abrasive materials used in practice.

It is very surprising that despite these findings, also supported by research activities aimed at the intentional disintegration of the sucked material [13,14], there are still some research activities considering the abrasive grain size as unchanged in the mixing and the subsequent focusing processes. Such studies, however, often draw incorrect conclusions, like it can be mentioned in [15], where authors correlate the acoustic signal with a number of impacting particles. The number of particles is calculated from the abrasive mass flow rate and the average size of particles entering the process, not from the average size of particles after the mixing and focusing processes. Nevertheless, experimental results published both in [9,10] make evident that the size of particles and their number substantially differ for experimental conditions applied by the authors of [15] and the abrasive material, which they used. The typical reduction of the abrasive particle size for experimental conditions introduced in [15] is 10 times, i.e., from approximately 200 μm to 20 μm. Therefore, the amount of the impacting particles is substantially higher than the one declared in [15].

Further research aimed at better precision of the AWJ machining, being in the progress recently, include not only cutting [16], which is still the predominant AWJ application. The progress in material engineering producing new hard-to-machine materials shifted the AWJ machining processes to the center of attention again [17,18]. Therefore, the new studies on AWJ turning [19,20], milling [21,22], drilling [23], and complex machining appeared [24]. Nevertheless, processes like grinding [25], piercing [26], or polishing [27] were also investigated throughout the last decade. Besides these AWJ applications, the micromachining [28,29] occurred, as a specific offshoot with some new and rather special problems related to the abrasive material and its behavior in the generation of the injection AWJ. All these applications of the injection AWJ are connected with the problem of abrasive disintegration during the mixing process and a subsequent focussing of the mixed jet. The behavior of the abrasive grains in these processes depends on the important abrasive material properties investigated already at the beginning of the 1990s [30].

The disintegration of concrete, rocks, and other brittle inhomogeneous materials by AWJ forms a significant special group of AWJ applications. However, in order to better identify the abrasive particles, metals were selected for the purposes of this communication. The abrasive particles etched into the surface of the cutting walls in metals are well identifiable. In spite of this, finding such a particle on the cutting wall often takes up to 30 min, because the entrapment of particles in the cutting micro-scratches on the kerf surface is not very frequent. Due to the different actions of particles on concrete or rocks, where the particles of the cut material are broken by abrasive grains rather than cut, the detection of any embedded abrasive particles is very unlikely.

The importance of considering the abrasive particle changes in the mixing process is evident from the broad impact of abrasive material on the resulting quality of the machined material surface. This statement is based on the research activities of many research teams all over the world. They are dealing with the influence of the abrasive particle shape [31], the abrasive material morphology, and/or mechanical properties of abrasive material [32]. Besides that, some works were focused on the experimental research of an abrasive impact on material [33], modelling of the particles’ energy [34], or on research of recycled abrasive particles and their efficiency [35,36]. Analyses aimed at the abrasive mass flow rate impact on the cutting system vibrations [37] and the respective cutting surface characteristics [38] are some of the attempts to prepare the models for a prediction of the surface roughness after the AWJ machining [17,39] or even to develop some systems enabling the online monitoring of surface roughness and topography [40].

The first aim of the study presented in this communication was to show that abrasive particles are less destroyed in the interaction with more ductile and plastic metals. This assumption has been partially confirmed. The second aim was to confirm, by observation of the abrasive particles etched into the cut material, the size of the particles after the mixing and focusing processes and their impact on the target material. The observations of cutting walls in several metal materials, presented in this contribution, bring some important findings supporting the necessity of the deeper studies aimed at the consideration of the disintegration of the abrasive particles in the mixing process. The most important observation is that the size of the particles ingrained into the cut walls in material ranges mostly between 10 and 20 microns.

## 2. Theoretical Background

The theory used for the calculation of the abrasive particle size after the mixing process in the cutting head of the injection AWJ has been presented namely in articles [10,11]. The particle size after the mixing process can be calculated from Equation (1):

(1) an = 24ρoEPao3coc24ρoEPao2coc + CDπaodo2μpo2o2γo2

Meaning of the respective variables is listed here: *a_o_*—the average size of the original abrasive particles, *a_n_*—average size of the abrasive particles after the mixing process, *c*—the speed of sound (mechanical vibration wave) in abrasive particle, *c_o_*—the speed of sound in liquid (usually water), *C_D_*—abrasive particle drag coefficient in liquid, *d_o_*—the liquid nozzle (orifice) diameter, *E_p_*—specific surface energy (energy for the creation of the new surface unit), *p_o_*—pressure before the liquid nozzle (pumping pressure), *ρ_o_*—liquid density (usually water density), *γ_o_*—compressibility factor (1 − *γp_o_*), *μ_o_*—nozzle discharge coefficient.

The theoretical relationship was supplemented by a correction taking into account the angle of entry of the abrasive material into the water jet passing through the mixing head. This angle causes the impact force reduction calculated from momentum change determined by Equation (2) presented firstly in [11]:

(2) $$\Delta \left(mv_{m}\right)=C_{D}\pi a_{o}^{3}\rho_{m}\frac{v_{o}^{2}}{c_{o}}-\frac{\pi a_{o}^{3}}{6}\rho_{m}v_{m}cos\alpha$$

The meaning of the respective variables is listed here: *a_o_*—the average size of the original abrasive particles, *c_o_*—the speed of sound in water), *C_D_*—abrasive particle drag coefficient in water, *m*—abrasive particle mass, *v_m_*—velocity of sucked abrasive material, a—angle between water jet and direction of the inlet particles velocity inside the mixing head, *ρ_m_*—density of abrasive material.

## 3. Studied Materials and Experimental Set-Up

Materials studied in this research phase are metals used in the Laboratory of Liquid Jet at the VSB—Technical University of Ostrava for about 15 years. The summary of their marking according to the three most common material norms is presented in Table 1. Each of the norms has been used in a particular part of the previous research and reflected the changes on market. The previous publications with identical metals were aimed at the product distortion in the AWJ cutting [41], the impact of the steel structure on the declination angle [42], investigation of the AWJ cutting forces [43], and the revised theoretical model [44]. The ČSN EN norm is transformed Czech national norm and it describes the metal type, its quality, and strength through the numerical code. DIN norm is the German norm describing metals by the combination of numbers and letters. The new German norm used for metal description is the W.Nr. norm (sometimes also referred to as W-Nr. or W.-Nr.). Metals are identified by five numbers with a dot between the first two ones. Almost all contemporary commercially available metals in Central Europe are described by this norm code.

All metal samples were plates 10 mm thick. The rectangle shape 60 mm × 10 mm was cut from each metal plate. These samples were presented in Figure 5 of the article [41]. The present observations were performed on the walls of the same samples presented in that publication. The standard abrasive material Australian garnet 80 mesh has been used for cutting. The cutting head Paser III^®^ (Flow International Corporation, Kent, WA, USA) was used for the cutting of samples. The traverse speeds were selected according to the material resistance in the AWJ cutting process. The respective traverse speeds were set so that the jet curvature inside the kerf was similar. Then the declination angle, the measure of cutting wall quality used in our laboratory, is similar for all samples, and also other quality measures, like the surface roughness and waviness, are comparable.

The traverse speed of 100 mm/min has been used for steels, 200 mm/min has been applied on copper and brass and duralumin has been cut with the traverse speed of 400 mm/min. Other parameters and factors important for the AWJ cutting are presented in Table 2 and they were identical for preparing all samples.

The average particle size after the mixing process in the Paser III^®^ cutting head (i.e., at the outlet from the focusing tube) determined for the experimental settings (Table 2) from Equations (1) and (2) is 24.95 μm (16.50 μm for the perpendicular inlet of particles). This value is approximately 10 times lower than the average particle size of the original abrasive material. Therefore, it was assumed that the particles observed on the sidewalls of cuts should be of similar size. Moreover, it was assumed that the size could be lower in the case of more resistant materials due to further disintegration of particles during the cutting process—interaction with the target material.

The sidewalls of the cuts and the etched abrasive particles were studied using Scanning Electron Microscope FEI Quanta 650 FEG (TermoFisher Scientific, Hillsboro, OR, USA). The observations are presented and described namely in Section 4. The settings of the microscope are presented in Table 3.

The element analysis aimed at the average atomic percentage is presented in Table 4 for both the original abrasive particles and the ones dug into the kerf walls (the used ones). The chemical and mineral compositions of the original abrasive material—Australian garnet (GMA 80 mesh) declared by the supplier (PTV s.r.o., Hostivice, Czech Republic, http://www.ptv.cz/abrasive/, 18 November 2021) are summarized in Table 5 and Table 6, respectively.

The element analysis presented in Table 4 corresponds with the chemical composition summarized in Table 5. Therefore, it is evident that particles dug or pressed into cut walls are particles of Australian garnet (GMA) used for the cutting of all metal samples.

## 4. The AWJ Cutting Walls Observations

This section summarizes namely the photos of the abrasive particles etched into the sidewalls of the kerfs cut in metal samples. The most important result of these observations is the presence of the visible abrasive particles dug into the walls. These particles indicate that the average grain size of the abrasive material interacting with the cut samples is close to the experimental results published and discussed in [9,10] and later theoretically justified in [11]. The approximate particle sizes have been measured on images from the electron microscope. The results of these measurements are summarized in Table 7.

**Figure 1:**
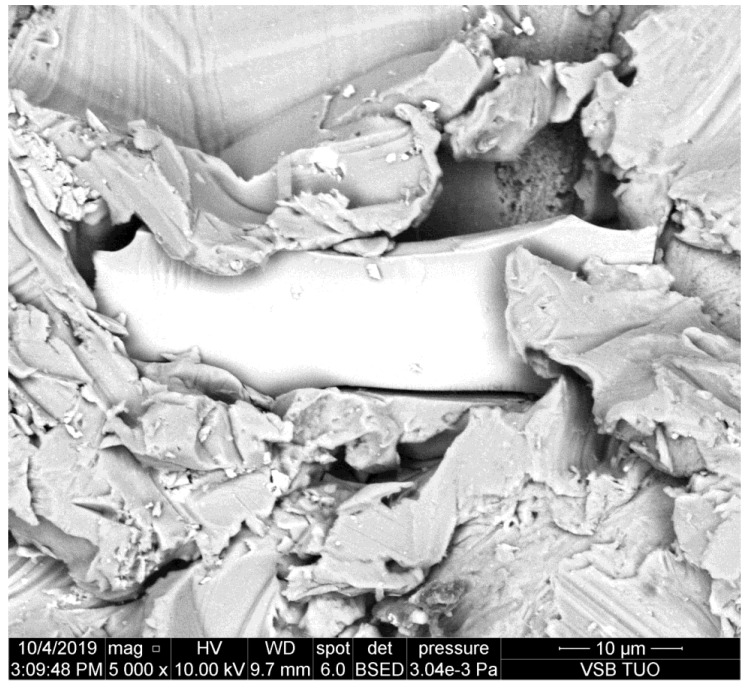
Abrasive particle pressed into the sidewall of the kerf made in duralumin.

The photo presented in Figure 1 shows the sidewall of the kerf produced in duralumin that is a rather mild metal and the plastic deformation is prevailing. Therefore, it is supposed that the abrasive particle is not significantly damaged by interaction with this metal.

**Figure 2:**
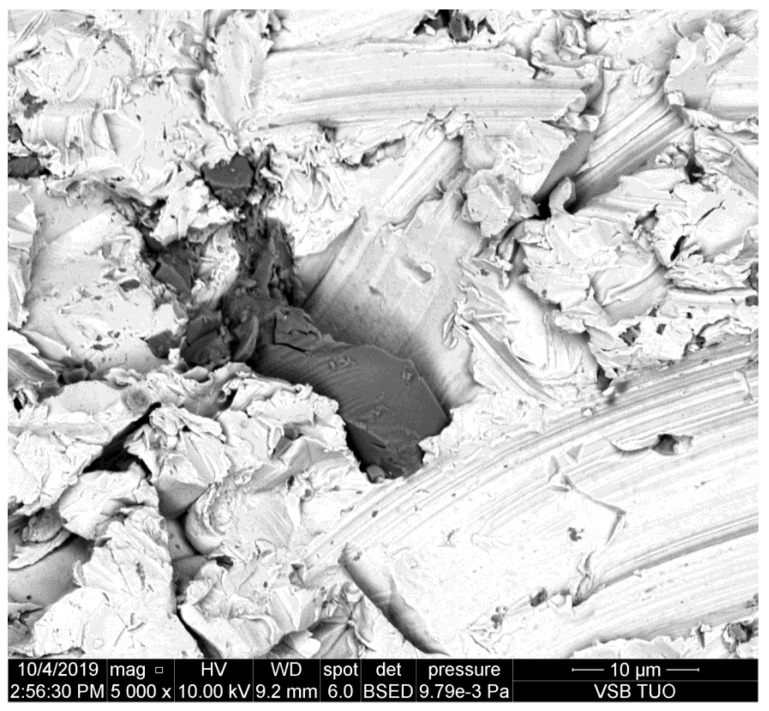
Abrasive particle impacted the sidewall of the kerf made in copper.

The metal presented in Figure 2 is copper. This is still quite mild metal with prevailing plastic deformation. It can be seen that the grain dug into the sidewall of the kerf is smaller than the one on duralumin (Figure 1). Some partial disintegration of the grain caused by the impact is evident.

**Figure 3:**
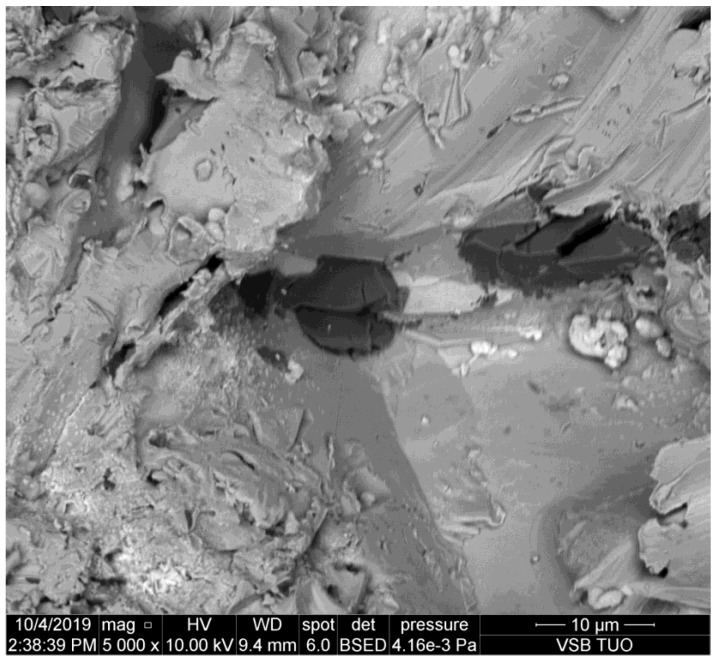
Abrasive particles observed on the sidewall of the kerf made in brass.

The kerf wall presented in Figure 3 is made in brass which is the more brittle metal. It can be observed, however, that the size of the abrasive particle pressed into the sidewall is similar to the previous case (copper). Simultaneously, it is evident that the damage of the abrasive grain is larger than in the case of more plastic metals like duralumin and copper.

**Figure 4:**
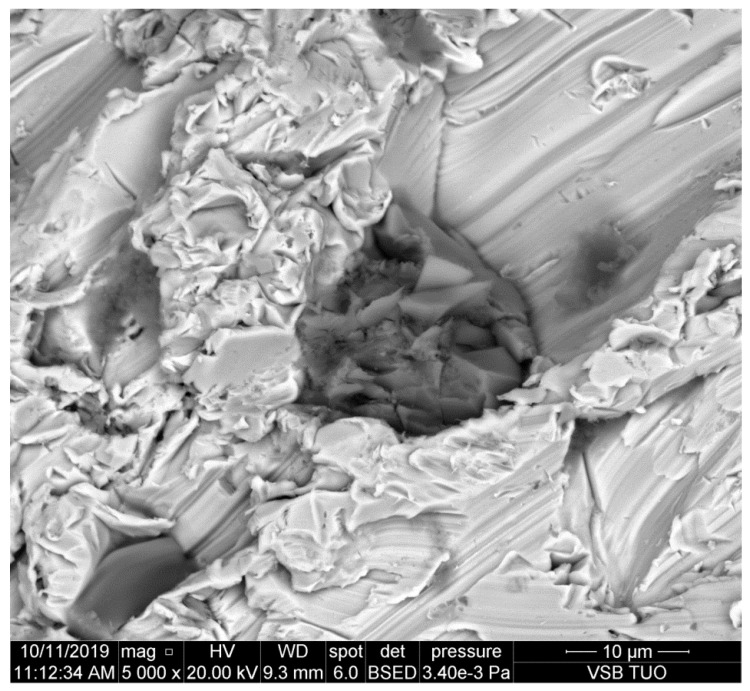
The abrasive particle pressed into the cutting wall of the common construction steel.

**Figure 5:**
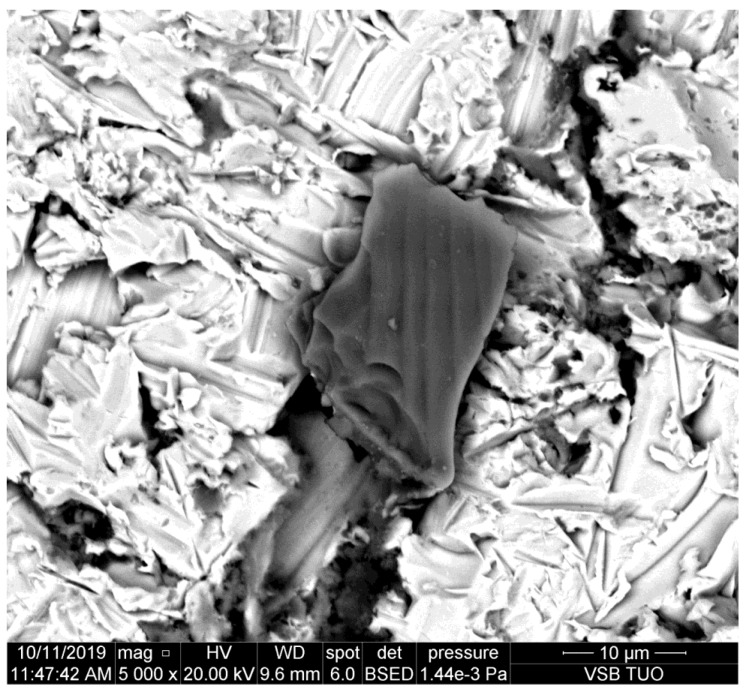
The abrasive particle detected in the cutting wall of the low alloy steel.

**Figure 6:**
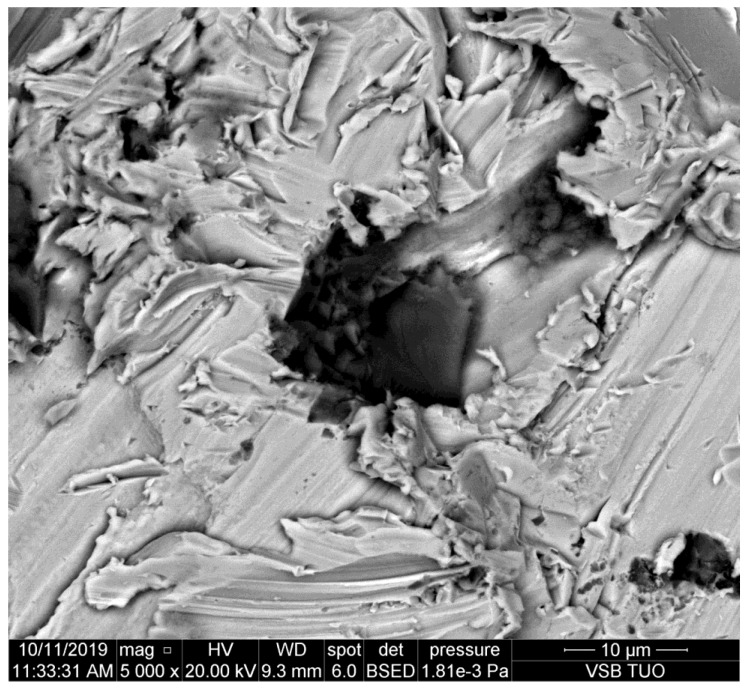
The abrasive particle embossed in the cutting wall of the high strength steel.

**Figure 7:**
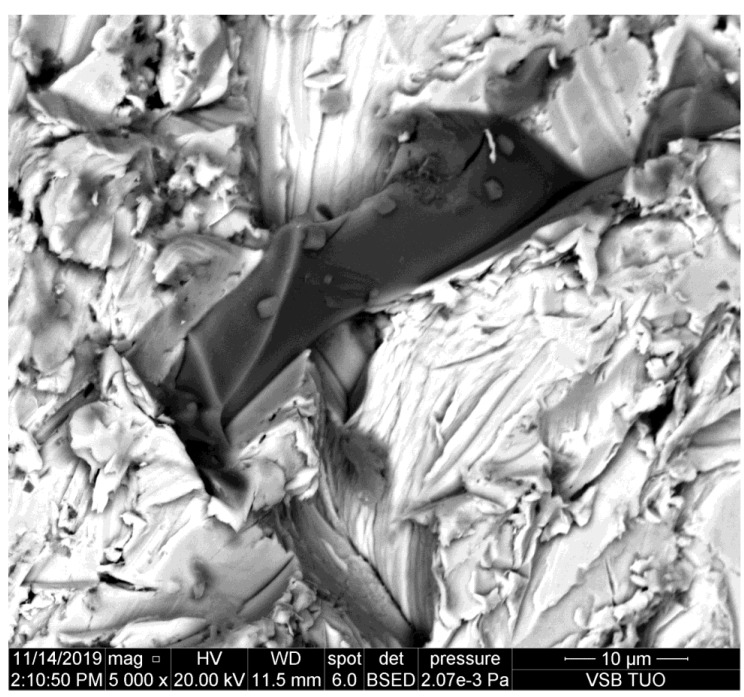
The abrasive particle observed on the cutting wall of the medium alloy steel.

**Figure 8:**
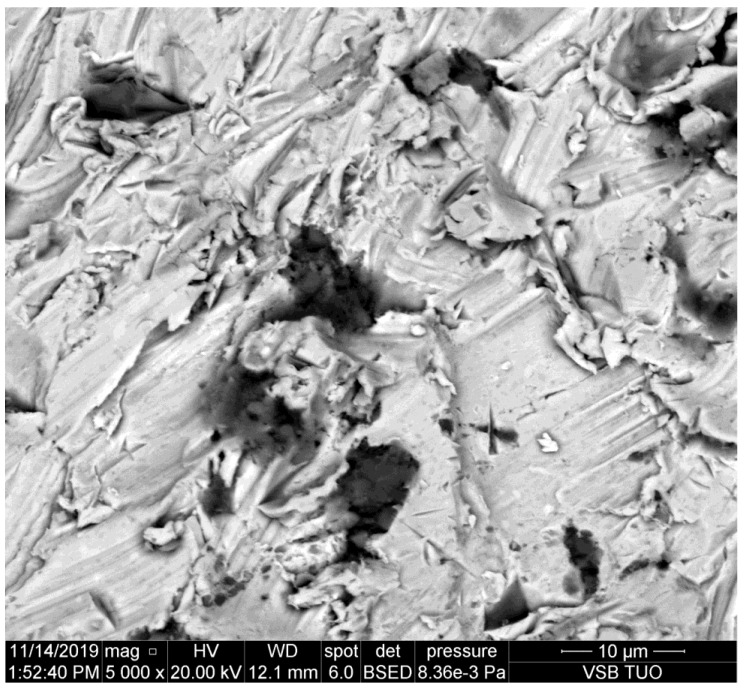
The abrasive particles pushed into the cutting wall of the tool steel.

Several kinds of steels are presented in Figure 4, Figure 5, Figure 6, Figure 7 and Figure 8. They are sorted according to the national standard (ČSN EN norm), which indicates the transition from the most plastic to the most brittle type by the second digit of the numerical code (the second column in Table 1). The footprints of the particles are typically scratches and furrows produced by ploughing of the abrasive particles. This observation corresponds with the assumption that kerf walls are created mainly by the so-called cutting removal of material, namely in the case of elastic-plastic and ductile materials. Exceeding the shear stress and strain is the most important factor in the case of metals. Some other materials could be disintegrated due to the different mechanisms—e.g., rocks and concretes, where the tensile strength plays an important role. The appearance of the kerf walls presented on scans from the electron microscope confirms, however, the cutting mechanism convincingly, namely on mild metals.

It can be observed that abrasive particles are usually seriously disintegrated by the impact on the material. However, their parts trapped in the kerf wall of the material indicate that the original size of them could be around 25 μm, as presented in [11]. Simultaneously, Figure 8 shows that the tool steel, the most abrasive jet resistant steel from the selected ones, causes the most serious disintegration of the abrasive particles. The size of the impacted rests of abrasive particles is below 10 μm on this steel.

## 5. Discussion

The study of the sidewalls of kerfs produced in various metals by the AWJ has shown some interesting results. The aim was to prove the assumption that the particle deterioration could be convincingly related to material characteristics like hardness, plasticity, brittleness, etc. However, this assumption is not proven sufficiently. Although some indicia, namely Figure 8, imply that abrasive particle damage can be more significant on harder and abrasive resistive metals, the amount of data is not sufficient for pronounced proving of such a statement. Exactly opposite, most of the observed particles impressed to the kerf walls indicate that the deterioration of the abrasive particles is not depending significantly on the material plasticity, hardness, and other characteristics influencing the cutting performance of the AWJ—it is evident also in Table 7, where the average size of particles detected on copper and brass is smaller than that on several plates of steel with higher strength and hardness and it is comparable with particles on high strength steel.

Nevertheless, the scans of the kerf walls with particles impressed to the material are showing that the most probable size of the cutting particles ranges between 10 and 30 μm even before their impact on the material. The same conclusion can be made based on the sizes of traces in the cut materials (see the chipping and scratches around the presented abrasive particles impressed to the kerf walls of the cut materials). This finding corresponds well with the conclusions of the previous experimental and theoretical research aimed at the disintegration of the abrasive material during the mixing process of the injection jets [9,10,11]. Those published research results showed convincingly that the most probable particle size after the mixing process is between 10 and 30 μm. The respective theoretical background is presented in Section 2 of this study. The usual settings of the AWJ, presented in Table 2, result in the average abrasive particle size after the mixing process of 16.50 μm (in the case of the perpendicular inlet of abrasive particles to the mixing chamber). In the case of the inclined inlet, like in the Paser III^®^ cutting head (the inlet angle is 45°) the average size increases to 24.95 μm. However, this value is still approximately 8–10 times lower than the size of the sucked abrasive particles (the average grain size ranges from 200 to 250 mm for garnet with sizing 80 mesh depending on how the average particle size is determined. Measurements in laboratories at the VSB—Technical University of Ostrava show the value 250 μm. Considering the inlet size 200 μm (usually presented as the equivalent for particles with size 80 mesh) the reduced average particles size of 10.70 μm is determined for the perpendicular inlet and the value 16.18 μm is calculated for Paser III^®^ head (with all other conditions identical).

Therefore, the abrasive particles observed on the sidewalls of the kerfs produced by AWJ can be either the particles produced in the mixing process, sometimes broken during the impact into a chain of smaller parts or they may be some partial parts of the greater particles. Nevertheless, the observed elements on the kerf walls seem to be at most ½ or ¼ of the interacting particles slipped during the ploughing of the cut material. In such a case, the size of the original particles is even closer to the theoretically determined values, i.e., around 25 μm [11]. This value was determined in the previous research aimed at the mixing process itself [11] performed with the identical experimental set-up as the one used for the preparation of samples used in the present experimental work.

The presented microscopic observation of walls of kerfs cut by the AWJ together with the previous experimental and theoretical results indicate that the particles interacting with the material after the mixing process are substantially smaller than the original ones. Therefore, their number is substantially higher, as it results from the mass conservation law. Omitting this fact leads to the invalid conclusions, as e.g., the source of the observed frequency in the article [15]. Because authors used almost identical set of the AWJ parameters and factors as presented in this study, the average particles’ size after the mixing process should be similar. Therefore, the frequency measured on the sample is hardly connected with the number of originally sized particles entering the mixing process, because particles are substantially disintegrated during the mixing process and only a very small amount of them could remain in the original size. Therefore, the correlation of frequencies generated on the cut sample with a number of particles determined from the abrasive mass flow rate and the original particle size is invalid.

The research presented in this study should support the need to consider the particle size change in each AWJ study, especially when measuring vibrations and acoustic signals. Frequencies measured on both the cutting head and the cut material should be correlated with the size changes of the abrasive particles. It is recommended namely in the case of the injection abrasive water jet, where the particle size changes are more dependent on the operating pressure and the cutting head configuration (including the ratio of orifice and the focusing tube size, the inlet of the abrasive position, and inclination and focusing tube length) than in the case of the slurry jet. The proper determination of both the particle size and the respective possible frequencies, caused by the AWJ machining, should help to monitor namely cutting of very hard and thick materials such as those studied in [17] or prepared for testing in [45]. Moreover, this monitoring could be useful also for online control in other AWJ machining processes, i.e., turning, milling, drilling, grinding, and polishing.

## 6. Conclusions

The microscopic study of abrasive particles etched to sidewalls of cuts produced in several metal types by AWJ brought these results:

- The size of the abrasive particles impressed to the kerf walls on studied metals varies particularly between 10 and 30 μm for the pumping pressure 380 MPa, the orifice diameter 0.25 mm, the focusing tube diameter 1.02, the stand-off distance 2 mm, the abrasive material Australian garnet 80 mesh, Paser III^®^ cutting head and the traverse speed causing the declination angle between 10° and 20°;
- (i.e., medium quality cut—the most usual in practice);
- The size of the abrasive particles impressed to the kerf walls seems to be little affected by material characteristics like hardness, yield strength, plasticity, etc., as it can be deduced from the average sizes of particles measured on the SEM images;
- The average size of abrasive particles impressed to the kerf walls differs very little from that determined after the mixing process of the injection AWJ, which can be considered as evidence that particle size is already substantially reduced during the mixing process and less by the cutting itself.

Based on these observations it is recommended to pay more attention to the mixing process and the product of mixing in studies of the AWJ machining processes. It should be further considered that the size of particles interacting with target material usually substantially differs from the ones of particles entering the mixing process.

## Figures and Tables

**Table 1:** Studied materials—summary of marking according to all norms used in previous published studies [41,42,43,44].

Material	ČSN EN Norm	DIN Norm	W.Nr. Norm
Construction steel K1	11523	St 52-3	1.0547
Low alloy steel K3	12050	C 45	1.0503
High strength steel K2	14220	16 MnCr 5	1.7131
Medium alloy steel K5	15142	42 CrMo 4	1.7225
Tool steel K4	19437	X210 CrW 12	1.2436
Copper	423001	E-Cu 57	2.0060
Brass	423223	CuZn40Pb2	2.0402
Duralumin	424201	AlCu4MgSi	3.1325

**Table 2:** The AWJ settings used in experiment.

Operating pressure	380 MPa
Stand-off distance	2 mm
Nozzle (orifice) diameter	0.25 mm
Focusing tube diameter	1.02 mm
Focusing tube length	76.2 mm
Applied liquid	water
Abrasive material	Australian garnet GMA
Abrasive sizing	80 mesh (0.25 mm) *
Abrasive mass flow rate	0.25 kg·min^−1^

* Explanation: Average grain size of Australian garnet 80 mesh has been measured in laboratories at the VSB—Technical University of Ostrava several times in the past on different measuring devices for particle size analyses (e.g., Fritch particle analyzer, ANALYSETTE 22, Fritsch GmbH, Idar-Oberstein, Germany, Malvern Instruments Ltd., Mastersizer 2000, Malvern, UK). The average value 0.25 mm has been determined and, therefore, it is used as the proven one, although some conversion tables present lower values (often close to 0.2 mm).

**Table 3:** Settings of the microscope FEI Quanta 650 FEG.

High voltage	10–20 kV
Magnification	5000
Detector	BSED (Backscattered Electron Detector)
Vacuum pressure	HiVac (10^−3^ Pa)

**Table 4:** Element analysis results—average percentage of the atomic weight from 10 measurements.

Sample	O	Si	Fe	Al	Mg	Ca	Mn	Ti
Original	65.41	12.48	8.55	8.91	3.77	0.65	0.23	-
Used	64.22	11.14	9.84	7.87	5.28	0.55	0.45	0.65

**Table 5:** Chemical composition of the original abrasive material according to the supplier’s list.

SiO_2_	Al_2_O_3_	FeO	Fe_2_O_3_	TiO_2_	MnO	CaO	MgO
36%	20%	30%	2%	1%	1%	2%	6%

**Table 6:** Mineral composition of the original abrasive material according to the supplier’s list.

Garnet	Ilmenite	Zirconium	Quartz (Free)	Other
97–98%	1–2%	<0.2%	<0.5%	<0.25%

**Table 7:** The average abrasive particle sizes detected on the cutting walls of particular metals measured on the microscope images using CorelDRAW^®^ Home & Student 2018 software (Corel Corporation, Ottawa, ON, Canada).

Metal Signature	Metal Kind	Average Particle Size (μm)
3.1325	duralumin	23.70
2.0060	copper	13.98
2.0402	brass	12.07
1.0547	construction steel	18.84
1.0503	low alloy steel	18.24
1.7131	high strength steel	14.58
1.7225	medium alloy steel	19.76
1.2436	tool steel	8.13

## Data Availability

Data is contained within the article.
